# Annotations

**Published:** 1910-04-23

**Authors:** 


### Annotations.

### Mortuary Accommodation in London.

If perseverance and persistence are of any avail, the Coroner for Southwark will no doubt some day attain his object of rendering the amenities of the Court at which he presides a credit instead of a disgrace to London. We have already on a previous occasion drawn attention to the installation in the City, for which Dr. Waldo is Coroner also, of an up-to-date cold-storage chamber for the preservation of bodies pending identification, and have dwelt upon the advantages of such a system, especially in cases of drowning, or rather of bodies found immersed, in the river. The same success which has eventually attended Dr. Waldo's representations to the City Fathers will no doubt in the end crown his efforts to arouse the Southwark Borough Council to a proper sense of its responsibilities; for the credit of the metropolis it is to be hoped that the much-needed reforms may be put in hand speedily, though, to judge by Press comments, it would appear that there is considerable local opposition which has first to be overcome. The outstanding facts of which some alteration is necessary are that at present the mortuary is half a mile from the Court House, and thus the jury have to walk a mile to view the body before the taking of evidence can begin. The route includes public houses, and the Coroner's officer has no statutory power to prevent the jury slaking a thirst on the way. In consequence it has happened that several members of a jury have returned from viewing the bodies of deceased persons in so hilarious a state that decency and decorum, let alone a properly considered verdict, have been quite unobtainable. Beyond this, the mortuary chamber itself is inadequately fitted for the purpose it is in use for. A new and well-equipped mortuary adjoining the Court is a crying need, and we trust Dr. Waldo will before long succeed in obtaining one.

### Medical Practice Abroad.

The General Medical Council has just issued a "Report as to the conditions under which medical and dental practitioners registered or legally qualified in their own country may practise abroad," in the shape of a shilling pamphlet, published by Spottiswoode and Co., Limited. Inquiries are often made at the Council Office and at the offices of medical journals, regarding the conditions under which a British diplomate may practise his profession in the Colonies or in a foreign country. Hitherto it has been difficult to give reliable information in all cases, as these conditions vary, and certainly nothing like any official pronouncement has as yet been placed before the profession. This pamphlet, which contains a vast amount of useful and, so far as we can judge, absolutely accurate information, will supply the want of such an official statement, and is to be cordially welcomed. It gives particulars of the conditions obtaining in all civilised countries, but is not quite complete as an exhaustive tabulation, since we miss British Borneo, China, and Rhodesia, for instance. The Council, however, distinctly refuses to accept responsibility for any inaccuracies or omissions that may be found, and states that the compilation is not to be regarded as official. At the same time it is a valuable little work, which should prove of decided usefulness. A cursory perusal shows the reader that throughout the British Empire reciprocity rules so far as the medical profession is concerned; that is to say, a practitioner registered in England can always succeed in establishing a claim to practise in a British dependency, subject to certain unimportant conditions. This is as it should be, but the medical profession is more than national, and we should like to see a rule made that every British subject who is a qualified medical practitioner, so long as he or she has obtained the qualification from a reputable source, should be allowed to practise in British dominions.

### The Nation's Children.

We have much pleasure in drawing attention to a useful and interesting booklet from the pen of Mr. Robert J. Parr, Director of the National Society for the Prevention of Cruelty to Children, on the subject of the nation's responsibility for its children. This little publication is "Wilful Waste," and the cheap price at which it is issued—6d.—places it within the reach of all who take an interest in one of the most important problems of the day. The scope of the booklet is well seen by a glance at the heading of the chapters. Thus the first is on Infantile Mortality, the second on Physically Defective Children, the third on Feeble-minded Children, while the following chapters deal with Pauper Children, Child Labour, Street Trading, Cruelty, and Responsibility respectively, each subject being dealt with in a simple, clear, and terse fashion, which extenuates nothing and sets down naught in malice. As a frontispiece, a series of characteristic photographs at once attract the reader's attention; and that these pictures are not wilfully chosen to portray the extreme, and therefore the non-representative, but are delineations of types, everyone who has had any experience of work among poor children can testify. In a few points we think Mr. Parr is perhaps too severe. For instance, something may be said in favour of child labour, and we are by no means at one with school teachers in their objections to the half-time system. Similarly we do not share Mr. Parr's views, without qualification, as to the inherent evil of street trading. We admit willingly that both these subjects stand in need of reform, supervision, and, above all, sympathetic treatment, and the more such views as the writer of this booklet holds are published and made known, the sooner will that reform be at hand. For that reason we welcome Mr. Parr's labour of love very cordially, and we should like to see it in the hands of every medical practitioner, clergyman, teacher, and last, but not least, every working-man. Our responsibilities with regard to the growing population are too serious to permit us to disregard the deplorable conditions under which pauper children exist at the present time.

